# Supplementary material for: Are carbon emissions trading and green financial instruments synergistic? -Comprehensive quantitative research based on content analysis
Source: PLoS One. 2024 Mar 7;19(3):e0298601. doi: 10.1371/journal.pone.0298601 (PMC10919601; doi:10.1371/journal.pone.0298601)
Supplement: S1 Data — (ZIP) [file pone.0298601.s001.zip › Data/地级市Z_F工作报告.zip!/░▓╗╒/╕╖╤⌠╩╨/└┤╫╘╣1⁄2╓┌║┼í╛┬φ┐╦╩2╛▌═°í┐_2014.docx]

各位代表：

现在，我代表市人民政府，向大会报告政府工作，请予审议，并请市政协委员和其他列席人员提出意见。

一、2013年工作回顾

过去的一年，在省委省政府和市委的正确领导下，全市上下认真贯彻落实党的十八大和十八届二中、三中全会精神，切实把扩大总量、提升质量摆在更加突出的位置，全面加快“四化”进程，全力促进经济社会又好又快发展，较好完成了市四届人大四次会议确定的目标任务。初步统计，全市生产总值突破千亿元大关，比上年增长12%左右；财政收入157.1亿元，增长15%；城镇居民人均可支配收入21430元，农民人均纯收入6810元，分别增长13%和15%。

一年来，主要做了以下几个方面的工作：

（一）注重总量扩张，全力促进经济提速发展。面对复杂形势和经济下行压力，认真落实中央和省各项稳增长政策，及时出台我市促进经济较快发展和大力发展民营经济一系列措施，有效保持了经济持续向好。深入实施工业强市战略，组织开展企业帮扶活动，实现规模以上工业增加值356亿元，增长14.8%。规模工业企业达到1066家，新增266家。私营企业和个体工商户分别增加到2.2万家和15.7万户。各类开发园区转型升级步伐加快，颍上、太和、临泉和界首扩区规划获得省政府批准。阜合现代产业园区起步区实现“七通一平”，福和家园安置小区投入使用，项目建设和招商引资工作取得明显成效。大力发展现代农业，粮食总产连续6年过百亿斤，实现“八连增、十连丰”，再次荣获全国粮食生产先进市称号。全市土地流转面积达353万亩，流转率42%，位居全省首位。经济作物面积扩大到440万亩，增长20％。肉蛋奶总量77万吨，规模养殖比重达74%。农产品加工产值550亿元，增长30%。省级以上农业产业化龙头企业发展到58家，农民专业合作组织4935家，注册家庭农场438户，新增省级现代农业示范区4个。森林增长工程扎实推进，成片造林12.7万亩，超额完成年度目标任务，受到省政府嘉奖。消费市场繁荣稳定，实现社会消费品零售总额516亿元，增长16.1%。金融保险业稳健运行，年末各类金融机构存款余额1751亿元，贷款余额788亿元，分别增长17.4%和22.5%。

（二）注重扩容提质，全面加快城乡建设步伐。围绕皖西北中心城市建设，一手抓外延扩展，一手抓内涵提升，组团式推进阜城大建设、大发展，全年完成基础设施投资77.7亿元，是上年的2.6倍。坚持规划引领，新一轮城市总体规划修编工作基本完成，统筹编制综合交通、市容环卫、市场建设等专项规划。万达广场、合肥百大等大项目落户我市，阜阳汽配城、农机大市场建成运营，临沂商城二期、四季青服装城和阜阳农产品中心批发市场等项目加快推进。高标准改造阜颍路、颍州路、北京路、阜王路等8条主干道路，三清路和北三环铁路立交工程竣工通车，城南新区路网、职教园区路网加快建设，三府街、三合巷、五院巷等20条小街巷改造完工。按照硬化、绿化、净化、美化、亮化和管理规范的要求，强力推进市容环境卫生、交通秩序、农贸市场“三项整治”，取得了历史性突破，切实改善了人居环境，提升了阜阳形象，得到广大市民的拥护和支持，受到省委、省政府的好评。依法实施控违拆违，基本实现违法建设“零增长”。大力开展砂石市场及运输秩序专项治理，荣获全省治超先进市称号。深入开展国家园林城市创建工作，全面实施城市增绿工程，新增绿地面积165万平方米。改革城市管理体制，实行市场化运作，建立了“属地管理、重心下移、分级负责、权责明确”的工作机制，入选全国智慧城市试点市。大力发展城市公交事业，新增100台空调公交车。高度重视保障性住房建设，开工建设3.4万套、基本建成1.8万套，超额完成年度目标任务。坚持安置优先，开工建设安置小区18个，建筑面积332.5万平方米。加大城乡统筹力度，县城和中心镇建设步伐加快，美好乡村规划编制工作加快进行，50个重点示范村建设成效明显。

（三）注重增强后劲，持续加强项目投资工作。坚持把扩大投资作为加快发展的第一抓手，切实加强项目工作调度，投资力度明显加大，全年固定资产投资690亿元，增长34%。昊源化工航天炉、东锦化纤和凯悦制药等重点项目建成投产，金种子生态产业园、开乐专用车三期、天泽业达等项目加快建设。阜六铁路建成运营，漯阜铁路复线电气化改造加快推进，阜新高速、102省道西湖至临泉段和202省道阜阳至阜南段、颍泉段建成通车。阜新高速临泉连接线等12条国省干线公路相继开工，建设里程达350公里。改造升级县乡公路214公里，加固整修农村公路危桥206座。沙颍河航道整治工程进展顺利，颍州港一期主体工程完工，全年港口吞吐量突破千万吨。阜阳机场旅客吞吐量35.7万人次，增长72.8%。切实加大招商引资力度，制定出台促进招商引资政策措施，在沿海发达地区设立招商办事处、选派招商小分队，成功举办了第六届华药会、第二届阜阳农博会等会展活动。全年引进市外项目1546个，实际到位资金665亿元，增长31%。大力开展政银企对接，加强金融战略合作，新增各类贷款144.5亿元，占年度计划的112%。

（四）注重改革开放，有效激发经济发展活力。坚持用改革的办法破解发展难题，扎实推进国有企业改革，市直医药企业改制工作顺利完成，华源纺织、阜阳轴承等企业重组工作取得积极进展。制定出台鼓励开展股权交易的政策措施，推动企业上市融资，贝克药业、金辉印务等9家企业列入省上市后备名单。进一步改革财政管理体制，实施“营改增”试点工作，全面推行国库集中支付制度和公务卡制度。加快地方金融体系建设，交通银行落户阜阳，颍淮、界首农村商业银行挂牌运营，颍上、界首、太和中银富登村镇银行正式营业。城南新区建设投资公司、市融资担保有限公司组建运营。认真落实结构性减税政策，全面清理涉企收费，实行工业投资项目和公益事业项目行政事业零收费，减免各项税费30亿元以上。全面清理行政审批事项，扎实推进“两集中、两到位”和并联审批工作，实施流程再造，市本级审批项目由245项减少到148项，精简幅度位居全省前列，经济发展环境明显改善。调整优化行政区划布局，顺利完成阜南县三塔集镇划归颍州区管辖工作。进一步规范招投标行为，建立了公共资源统一交易平台。深化医药卫生体制改革，全面推进县级公立医院改革。加快文化体制改革步伐，再次荣获全国文化体制改革先进地区称号。大力发展对外贸易，实现进出口总额13.7亿美元，增长24.5%；外商直接投资1.3亿美元，增长24.9%。

（五）注重民生改善，统筹发展社会各项事业。始终坚持以人为本、民生为重，33项民生工程全面完成。深入实施居民收入倍增计划，提高了城乡最低生活保障标准和最低工资标准，实现困难群体临时救助制度城乡全覆盖，居民消费价格涨幅控制在2.2%。认真做好就业再就业工作，成功举办“接您回家”系列活动，新增农村劳动力转移就业13.7万人，城镇登记失业率控制在4%以内。进一步扩大社会保险覆盖面，5项社会保险超额完成年度任务，阜城基本医疗保险实现市级统筹。大力推进创新体系建设，实现高新技术产业产值170亿元，增长30%，发明专利授权量128件。校舍安全工程和学前教育三年行动计划全面完成。阜城基础教育设施三年提升计划和阜阳医疗资源建设三年发展计划首战告捷，累计完成投资19亿元，阜阳三中新校区投入使用，市人民医院新区、安医大附属阜阳医院等项目进展顺利。扎实推进职教大市建设，阜阳技师学院主体工程建成使用。深入实施文化惠民工程，支持新闻事业发展，加强对外宣传工作，阜阳知名度和美誉度得到提升。广泛开展全民健身活动，成功举办市第十二届运动会。全面加强精神文明建设，全市67人入选中国好人榜，马虎被评为第四届全国道德模范。加快发展旅游业，八里河景区被评定为国家5A级旅游景区。加强生态文明建设，耕地保护目标顺利实现，节能减排任务较好完成，国家级再生材料质检中心落户阜阳，界首市入选首批国家循环经济示范县。坚持计划生育基本国策，努力稳定低生育水平。组织实施新一轮扶贫开发攻坚规划。妇女、儿童及老龄、老干部工作进一步加强。红十字、社会慈善及残疾人事业稳步发展。

（六）注重和谐稳定，深入推进社会治理创新。加强和改进新形势下群众工作，坚持领导开门接访、带案下访、包案约访，进一步畅通信访渠道，依法规范信访秩序，一批信访积案得到化解。全面推行重大事项社会稳定风险评估制度，深入开展矛盾纠纷排查调处工作，努力解决群众关心的热点、难点问题。强化食品、药品和生产安全监管，全年未发生重特大安全事故。进一步完善应急管理体系，突发事件处置能力逐步增强。深入推进法治阜阳、平安阜阳建设，组织开展严打专项行动，社会治安形势持续好转。支持驻阜部队建设，武警、人防和民兵预备役工作进一步加强，再次荣获省双拥模范城称号。有序推进基层社区建设与管理。民族、宗教、统计、文物、档案、地方志、地名、气象、地震、外事、侨务、港澳台等工作也都取得了新的成绩。

过去的一年，我们还注重加强政府自身建设，认真开展兴学风、转作风、清政风、树新风“四风行动”，坚持市政府常务会议学法制度，修订完善了市政府工作规则。自觉接受人大法律监督和政协民主监督，认真执行人大决议、决定，办理人大议案和建议73件、政协提案267件。深入推进政务公开，加强电子政务和政府网站建设，扎实做好“12345”市长热线和网络问政工作。实施了市直机关公务员首批交流轮岗，开展了市直行政事业单位银行账户及土地房产“双清”工作。严格执行中央八项规定、国务院“约法三章”和省市相关规定，切实精简会议、文件，开展公务用车专项治理，清理规范节庆、论坛、展会和各类检查评比活动，“三公”经费下降23%，政风建设取得新成效。

在肯定成绩的同时，我们也清醒地看到，我市经济社会发展中还存在不少困难和问题。一是经济总量不大、发展不快、结构不优的问题依然突出，人均水平偏低，工业化、城镇化水平不高。二是投资规模偏小，招商引资总量不大，新兴产业发展不足，非公经济发展不快，部分企业经营困难，财政收支平衡压力较大。三是土地、资金、水资源等要素制约日益突出，节能减排和生态环境建设压力不断增大，转变发展方式任务艰巨。四是社会事业发展滞后，公共服务均等化水平较低，社会治理工作面临新的挑战。同时，一些政府部门和工作人员思想解放不够、工作作风不实、执行落实不力等问题仍较突出，形式主义、官僚主义、享乐主义和奢靡之风还不同程度存在，发展环境有待进一步优化。对此，我们一定高度重视，采取有力措施，认真加以解决。

各位代表！过去的一年，我们能够战胜各种困难和挑战，取得来之不易的成绩，是省委、省政府和市委正确领导的结果，是市人大、市政协大力支持的结果，是全市广大干部群众锐意进取、奋力拼搏的结果。在此，我代表市人民政府，向广大工人、农民、知识分子、干部、离退休老同志，向驻阜解放军指战员、武警官兵和政法干警，向各民主党派、工商联、无党派人士、各人民团体和各界人士，向中央和省驻阜单位及所有关心支持阜阳建设与发展的同志们、朋友们，表示衷心的感谢和崇高的敬意！

二、2014年工作安排

今年是深入贯彻落实党的十八届三中全会精神、全面深化改革的开局之年，也是完成“十二五”规划目标任务的关键一年。我们虽然面临宏观形势偏紧、结构矛盾突出、要素制约明显、区域竞争加剧等困难和挑战，但也有着全面深化改革红利集中释放、沿海发达地区产业加速转移、新型城镇化战略加快实施、中原经济区建设深入推进、扶持皖北力度持续加大、智慧城市试点工作全面启动等诸多机遇。同时，我市人力资源充足、自然资源丰富、交通区位优越、市场潜力巨大，政治生态、发展环境明显优化，广大干部群众干事创业的氛围日益浓厚，阜阳正处于大有可为的黄金发展期。只要全市上下振奋精神，团结一心，开拓进取，扎实苦干，就一定能够夺取经济社会发展的全面胜利！

今年政府工作的指导思想是：全面贯彻落实党的十八届三中全会、市委四届六次全会和全市经济工作会议精神，坚持“稳中求进、进中求好、好中求快”工作总基调，以全面深化改革为根本动力，以扩大有效投入为主攻方向，大力推进城镇化、工业化和农业现代化进程，强力推进项目建设，着力转变发展方式，全面激发市场活力，切实保障和改善民生，促进经济持续健康发展、社会和谐稳定。综合各种因素考虑，主要预期目标是：全市生产总值增长11%以上；财政收入增长14%；固定资产投资增长30%以上；社会消费品零售总额增长14%；进出口总额增长20%；城镇居民人均可支配收入、农民人均纯收入分别增长12%和14%；居民消费价格涨幅控制在3.5%左右；城镇登记失业率控制在4%以内；完成节能减排年度任务。

实现上述目标，重点抓好以下八个方面的工作：

（一）着力深化重点领域改革。全面落实中央和省改革总体部署，坚持以经济体制改革为重点，紧紧围绕市场在资源配置中起决定性作用和更好发挥政府作用，以更大决心和勇气全面推进改革。一是深化行政体制改革。进一步加大简政放权力度，坚决落实好中央和省取消、下放的行政审批事项，编制行政审批目录清单，公开接受社会监督。深化人事制度改革，有效实施部门改革，合并调整相关机构，优化部门服务职能。加快推进事业单位分类改革，推动行业协会、中介服务组织与行政机关脱钩。二是深化农村综合改革。坚持和完善农村基本经营制度，认真做好颍泉、临泉省级农村综合改革示范试点工作，扎实推进土地承包经营权确权登记颁证，建立健全土地承包经营权流转激励机制和风险防范机制，按照规范化、契约化、法制化要求，积极引导土地向新型农业经营主体流转。加强农村集体资金、资产、资源管理，创新公共基础设施建设和管理机制。建立健全农业贷款和农业保险制度，稳步开展土地承包经营权和宅基地使用权抵押贷款、租赁、调换试点工作，探索建立农村多元统一的土地市场。三是深化金融综合改革。鼓励引导金融机构开展产品创新、服务创新，满足多元化的信贷需求。加强资本运作，努力提高直接融资比重。加快阜南、临泉中银富登村镇银行筹建工作，力争实现村镇银行县域全覆盖，确保太和、临泉、阜南、颍上农村信用联社上半年完成改制任务，引导更多资金投向小微企业、涉农组织和县域经济。四是深化财税体制改革。进一步理顺市、区财政体制，完善县级基本财力保障机制，加大税收征管力度，确保各项收入应收尽收，促进财政收入持续较快增长。改进预算管理制度，压缩一般性支出，年内实现县级以上部门预算全部公开。继续做好“营改增”扩围试点，大力推进政府向社会力量购买服务。五是深化国有企业改革。坚持着眼发展、循序渐进、规范操作、确保稳定，以发展混合所有制经济为主攻方向，按照“产权多元化、主业规模化、管理现代化”的要求，统筹运用战略合作、资产重组等方式，因企施策、分类推进，积极有效促进我市国有企业改革工作。

（二）着力扩大有效投资规模。更加注重发挥投资对经济发展的关键作用，坚持经济工作项目化、项目工作责任化，努力实现全年固定资产投资有一个大的突破。一是加强项目谋划工作。围绕国家产业政策和投资导向，坚持以资源换项目、以市场换项目，谋划编制一批重大项目，实现储备项目投资额5000亿元以上。加强项目申报推介和招商工作，形成滚动增加、梯次推进的良性循环。二是健全项目推进机制。建立完善项目调度制度，对重点项目坚持“定投资主体、定牵头领导、定完成时限、定工作责任”，强力加以推进。进一步加强部门联动配合，畅通重点项目“绿色通道”，坚决破除项目实施中的各种障碍和瓶颈，努力营造项目建设的浓厚氛围。修订完善项目建设考核办法，以项目看实绩，以项目看水平，以项目看担当，严格兑现奖惩。三是拓宽项目投资渠道。坚持多措并举筹集发展资金，采取土地出让变现、开展银企对接、争取政策资金、激活民间投资等方式，努力盘活存量资金、扩大增量资金。突出招商引资主渠道作用，抢抓沿海发达地区“腾笼换鸟”产业转移机遇，加大政策引导和奖励力度，推进领导招商、部门招商、专业招商、产业招商和以商招商，切实提高招商实效，努力扩大引资规模，全年到位市外资金增长40%以上。坚持以质量和效益为中心，统筹引资、引技与引智，注重挑商选资工作，坚决摒弃拼土地、拼资源、拼环境，坚决防止引进落后产能和污染企业。

（三）着力推进新型城镇化进程。围绕建设皖北区域性中心城市，坚持以人为核心的城镇化，大力度改变城镇形象、高标准完善城镇功能、全方位发展城镇产业、精细化加强城镇管理、真感情惠及城镇民生，全面加快城乡建设步伐。一是高起点完善城镇体系。坚持大阜城构想、大手笔谋划，立足打造豫皖城市群首位城市和综合交通、商贸物流、教育医疗等区域中心，高标准编制控制性详规和各类专项规划，优化城市布局，拓展发展空间。按照中等城市标准，高起点规划建设颍上、临泉、太和、阜南县城和界首市。加快推进一批特色中心镇建设，认真抓好美好乡村示范点，全面推进中心村建设，加快构建阜阳区域中心城市、县城中等城市、特色乡镇、美好乡村“四位一体”的城乡发展体系。二是高标准做大城市规模。坚持大项目支撑、大力度推进，春节后阜城集中开工一批重点基础设施项目。加快城南新区建设，打造新型城镇化示范区。扎实推进颍河综合治理、三角洲东岸人居环境整治等项目建设，重点实施256省道阜城西南大外环、东三环路及颍河大桥、职教园区路网、颍上南路和阜王路南延工程。加快102省道阜阳至颍上段、340省道阜阳至杨楼孜段和沙颍河航道整治、南照淮河取水、颍州港工程建设进度，力争阜阳至淮滨高速、商阜杭高铁早日开工。研究编制阜阳市域及与周边城市城际铁路布局方案。三是高品位提升城市内涵。按照新型城镇化要求，注重强化产业支撑、带动人口集聚，统筹发展资本密集型、技术密集型和劳动密集型产业，大力发展就业容量大的服务业，促使城镇与产业发展良性互动。注重完善公共服务，规划建设市博物馆、规划馆、档案馆、文化馆和政务服务中心，开工建设6.3万套保障性住房，全面启动首期12平方公里棚户区改造工程。注重就地城镇化，有序推进农业转移人口市民化，研究制定户籍制度改革方案，全面推行居住证制度，努力解决进城农民工家庭留得下、住得稳、生活得好的问题。注重提升城市形象，按照“五化一规范”要求，坚定不移地把“三项整治”工作向纵深推进，全面提升城市管理水平。加强园林绿化工作，大力支持文化生态旅游产业发展，争创国家园林城市。加快发展公交事业，今年再增加100辆空调公交车，推动城区公交线路向周边乡镇延伸。通过不懈努力，把阜城打造成为皖北地区美丽宜居、文明和谐的中心城市。四是高强度破解瓶颈制约。坚持安置优先，依法做好土地房屋征迁工作，确保城市建设顺利进行。切实加大控违拆违力度，始终保持高压态势，确保违法建设“零增长”。着眼求增量、盘存量、调结构、提效益，扎实推进农村土地综合整治和城乡建设用地增减挂钩，争取开展“人地挂钩”试点，努力拓展用地空间。注重发挥城市投融资平台作用，积极发行市政债券，进一步拓宽融资渠道。

（四）着力实施工业突破。牢固树立工业立市、工业强市理念，大力实施工业突破战略，全年规模工业增加值增长16%以上。一是突出转变方式，不断提升工业经济规模和水平。按照新型工业化要求，大力实施创新驱动战略，重点培育壮大生物医药、电子信息等战略性新兴产业，努力打造华东地区最大的生物医药生产基地。运用先进技术改造传统产业，做大做强专用汽车、煤化工及特色纺织产业。加大工业招商力度，引导企业采取资产重组、战略合作、产业链招商等方式，引进一批关联性强的工业项目。大力推进工业化、信息化深度融合，加强智慧城市建设，采取市场化、社会化运作模式，实施物联网、云计算中心等重点项目，以此催生新兴产业、推动产业发展。深入开展与央企、全国知名民企和皖粤对接合作，建立健全沟通协调机制，促进更多的大项目、好项目落户阜阳。坚持“两个毫不动摇”，切实把民营经济作为工业发展的重要抓手，认真落实鼓励民间投资的扶持政策，放宽经营条件，着力打破“玻璃门”、“弹簧门”、“旋转门”等障碍，激发民间投资活力。二是突出扶优扶强，努力做大支柱产业和骨干企业。注重挖掘现有企业潜力，健全工业发展推进机制，出台扶持政策，不断壮大八大工业支柱和骨干企业，努力打造千亿元产业，培育百亿元企业。加快阜阳煤基新材料、国机集团轴承、欣奕华新材料和金种子生态产业园建设，有效扩大刘庄、谢桥和口孜东煤矿产能，实施阜阳卷烟厂和华润啤酒、华源纺织公司退城进园，推进江汽集团改装车底盘制造基地、开乐专用车三期、华鑫集团“双改双百”等重点项目建设，支持贝克药业、华信药业、金辉印务等企业上市融资，扶持壮大小微企业，全年新增规模工业企业200家以上。三是突出扩区升级，切实增强开发园区承载力。按照产城一体要求，进一步加大投入，加强基础设施建设，加快培育特色产业，形成一批主业突出、特色鲜明、核心竞争力强的开发园区。进一步优化园区功能布局，严格执行投资强度、亩均税收、建设容积率等政策规定，依法清理闲置和低效利用土地，提升园区发展的集聚度和集约化水平。加快园区转型升级步伐，支持市开发区升级为国家级开发区，认真开展颍东、颍泉、颍州和阜南园区扩区升级工作。支持阜合现代产业园区加大引资力度，加快项目建设，启动拓展区土地征迁、道路建设工作，努力打造改革开放的引领区、产城一体的先行区和社会管理的示范区。

（五）着力提升农业现代化水平。牢固树立经营农业理念，紧紧围绕“规模、结构、转化、合作”，以市场为导向、以科技为手段、以改革为动力，促进农民增收、农业增效。一是切实加快现代农业发展步伐。深入实施粮食生产“四大行动”，大力建设高标准农田，依靠科技，提高单产，稳定总产，为国家粮食安全作贡献。依托市场，调整结构，提高效益，为农民增收找出路。大力发展特色农业，着力扩大林果花卉苗木、中药材等高效经济作物种植面积，积极开展国家和省级现代农业示范区创建工作。引导阜城三区重点发展城郊型农业，在县城周边规划建设一批具有综合功能的农业园区。加快推进新一轮“菜篮子”工程，加强蔬菜生产基地建设。实施畜牧业升级计划，促进畜牧水产规模化养殖。高度重视农产品质量安全，强化从田间到餐桌全过程监管，确保人民群众“舌尖上的安全”。二是大力构建新型农业经营体系。加快培育新型农业经营主体，加大对家庭农场的定向扶持，支持种养大户成立家庭农场。积极发展股份合作、土地合作等新型合作，促进农民合作社持续健康发展。加强农产品加工示范基地建设，打造粮食、蔬菜、肉类等产业集群，做大做强龙头企业，推动农产品加工向精深化、品牌化和集群化发展。通过富裕农民、提高农民、扶持农民，让农业经营有效益，让农民成为体面的职业。三是不断增强农业综合生产能力。深入推进小型农田水利重点县建设，实施全省小型水利工程改造提升“5588”行动计划，新建高标准基本农田93万亩。扎实推进农村公路“村村通”延伸工程，加强电力基础设施建设。加快构建新型农业科技服务体系，实施农机化推进工程，不断提高农业社会化服务水平。

（六）着力加强商贸名城建设。围绕建设现代商贸物流中心，加快培育大市场、发展大贸易、搞活大流通，不断提升服务业发展规模和水平。一是努力扩大消费需求。抓住国家放开服务业准入限制和“营改增”扩围的有利时机，不断改善消费环境，积极发展健康养老、旅游休闲、体育健身等生活性服务业，加快发展研发设计、信息服务、电子商务等生产性服务业。大力推进流通体系建设和流通产业发展，加强仓储中心、配送中心建设，促进消费稳步增长，推进服务业提速提质提效，充分发挥消费的基础作用。加强市场调控，保持价格总体水平基本稳定。二是加快商贸市场建设。深入实施城市商业网点规划，推进专业市场和商贸购物中心建设，积极引进大型城市综合体，提升人民路和西二环路核心商圈辐射功能。加快推进万达广场、宝龙城市广场、元一时代广场和临沂商城二期、阜阳农产品中心批发市场等重点项目建设，支持汽贸物流园创建国家级重点商贸物流园。三是大力发展对外贸易。实施外贸企业孵化工程，加强太和医药中间体研发、香料项目扩建和国家级发制品转型升级示范基地建设。壮大黄岗柳编等产业集群，争创国家重点出口基地。扩大纺织服装、中药材、洁具等优势产品出口，进一步提高经济外向度。

（七）着力加快县域经济发展。县域经济薄弱是制约我市经济发展的重要因素，必须摆在更加突出位置，不断强化推进措施，全面提升县域经济综合实力。一是立足争先进位，锁定发展目标。组织开展“县域经济突破年”活动，制定落实加快县域经济发展的政策措施，修订完善考核办法，加强分类指导，切实解决发展中的困难和问题，全面掀起县域经济发展热潮。各地要拔高标杆，研究确定争先晋类目标。二是坚持因地制宜，推动竞相发展。立足区位条件、资源禀赋和产业基础，坚持“双轮驱动”、“四化”同步，做到以农业现代化为基础、工业化为重点、城镇化为保障，着力打造劳动密集型、资源转化型、市场依托型、承接转移型产业基地，走出一条一、二、三产齐头并进、协调发展的路子。切实把扩大有效投入作为强力支撑，全面加强城镇道路管网、电力通讯等基础设施建设，着力完善学校、医院等公共服务设施，大力引进城市综合体、大型房地产和大市场等项目，迅速壮大县域经济实力和规模。支持引导颍上县在做大煤炭产业的同时大力发展非煤工业和旅游产业，界首市重点发展循环经济，太和县优先发展生物医药产业，阜南县加快发展纺织服装和农副产品加工业，临泉县注重发展机械电子和农副产品加工业。三是推进全民创业，促进共同富裕。切实把全民创业作为加快发展的强大动力，在开发园区设立创业园和孵化器，大力支持返乡农民工、高校毕业生、企业下岗人员等群体自主创业。进一步降低门槛，鼓励社会资本进入基础设施、基础产业、公共服务等领域，着力营造宽松的创业环境，激发全社会的创造活力。同时，抢抓大别山片区扶贫开发机遇，大力实施整村推进、产业化扶贫和“雨露计划”，打好新一轮扶贫开发攻坚战。

（八）着力改善民生促进和谐。按照“科学规划、全力服务、加大投入、守住底线”的要求，大力推进基本公共服务均等化，让发展成果更多更公平地惠及全体人民。一是切实保障和改善民生。按照省政府统一部署，明确民生工程内容，落实配套资金，加快实施进度，切实提高工程质量和效益。坚持把稳定就业作为经济发展的优先目标，重点做好农村劳动力、高校毕业生、城镇就业困难人员的就业工作，动态消除“零就业”家庭。进一步深化“接您回家”活动，制定落实扶持政策，鼓励农民工返乡创业，引导农民工就地就业。着眼保基本、兜底线、促公平、可持续，进一步完善社会保障体系，切实加大社会救助力度，全面做好各项民生保障工作。二是统筹发展各项社会事业。加大政府科技投入，培育高新技术企业，促进科技成果转化和运用。坚持长远抓教育、抓长远教育，深入实施阜城基础教育设施三年提升计划，开工建设阜阳第七高级中学。大力推进教育体制综合改革，促进义务教育均衡发展。进一步整合职业教育资源，加快职教园区建设步伐，积极创建阜阳幼儿师范高等专科学校。深入推进阜阳医疗资源建设三年发展计划，深化医药卫生体制改革，巩固基本药物制度，提高基本医疗、公共卫生保障和服务水平。深入开展群众性体育活动，积极备战省第十三届运动会。全面推进文化改革创新、繁荣发展，办好“颍淮群众文化大舞台”，加快发展文化产业。编制全市旅游发展规划，加大宣传推介力度，提升旅游产业辐射力和竞争力。实施计划生育“单独两孩”政策，加大奖励扶助政策落实力度，稳定适度低生育水平。认真实施新一轮妇女儿童发展规划纲要。加快推进老龄事业和养老产业发展。三是大力加强生态文明建设。加大节能减排工作力度，开展“颍淮蓝天工程”大气污染防治行动，强化工业废气、机动车尾气、城市扬尘、秸秆焚烧治理，大力淘汰“黄标车”。加快建设颍州、泉北污水处理厂，加强乡镇污水处理设施建设。深入实施森林增长工程，持续开展以“三线三边”为突破口的城乡环境综合治理，扎实推进卫生创建活动。切实加强耕地保护，确保面积不减少、质量不下降。加快治淮重点工程建设，改善境内河流水质，加强茨淮新河水源地保护。四是全面提高社会治理水平。严格落实信访责任制，推进依法治访、访诉分离。建立健全社会稳定风险评估机制，畅通和规范群众诉求表达与权益保障渠道。深入推进法治阜阳、平安阜阳建设，强化食品、药品和生产安全监管，有效防范和坚决遏制重特大事故的发生。加强应急平台和专业队伍建设，提高应急保障能力。进一步完善社会治安防控体系，严厉打击各类违法犯罪活动，提升人民群众安全感和满意度。推进城乡社区标准化建设，做好第九届村委会换届工作。强化统计工作基层基础，认真开展第三次全国经济普查。加强国防动员、后备力量建设和人民防空工作，支持驻阜部队现代化建设，积极争创全国双拥模范城。高度重视国家安全、保密工作。支持工会、共青团、妇联和关工委、老龄委工作。做好档案、地震、气象、地名、地方志和民族、宗教、外事、侨务、港澳台等工作。发展红十字、残疾人和福利慈善事业。

三、加强政府自身建设

面对新形势、新任务，政府使命在肩、责任重大。各级政府必须切实加强自身建设，加快推进治理体系和治理能力现代化，着力提升科学执政、民主执政、依法执政水平。

（一）全面加强学习，提升科学施政能力。切实把学习作为提高素质、增强本领、做好工作的根本途径，深入学习党的十八届三中全会和习近平总书记系列讲话精神，主动学习政策、研究政策、用好政策，善于把上级精神、外地经验同本地实际有机结合，努力使我们的思想更加适应形势发展、知识更加适应时代要求，不断提高研判形势、抢抓机遇和驾驭市场经济的能力。进一步增强进取意识、机遇意识、责任意识和忧患意识，切实强化问题导向、底线思维，善于观大势、谋大事，自觉在大局下想问题做工作，以自我革新的勇气和胸怀，敢于啃硬骨头，敢于涉险滩，拿出更大决心冲破思想观念障碍，寻求改革共识的最大公约数，汇聚加快发展的强大正能量。

（二）正确履行职能，提升依法行政能力。加快转变政府职能，加强公共服务、市场监管、社会管理、环境保护等职责，切实把工作重点转到创造良好环境、提供优质服务、维护公平正义上来，不断创新公共服务供给方式，满足群众多元化服务需求。始终坚持依法行政，带头依法办事，切实规范行政执法行为。坚持依法科学民主决策，完善规范性文件、重大决策合法性审查机制，把公众参与、专家论证、风险评估、集体讨论决定作为必经程序，提高决策透明度和群众满意度。进一步深化政务公开，继续办好市长热线电话，加快推进网上审批。各级政府及其工作人员要主动接受人大法律监督和政协民主监督，认真办理人大代表议案、建议和政协委员提案，广泛听取各民主党派、工商联、无党派人士和各人民团体的意见，鼓励支持人民群众、新闻媒体对政府工作进行监督。

（三）坚持真抓实干，提升执政为民能力。认真组织开展党的群众路线教育实践活动，全面深化“四风行动”，始终把促进社会公平正义、增进人民福祉作为出发点和落脚点。各级政府及其工作人员要深入基层、深入一线，问政于民、问需于民、问计于民，把更多的精力投入到解决人民群众关心的问题上来。牢记“实干兴邦、空谈误国”，切实拿出“踏石留印、抓铁有痕”的劲头，鼓起“逢山开路、遇水搭桥”的精神，敢于担当、勇于负责，下决心解决决策不敢拍板、矛盾不敢面对、工作推动不力等影响发展的突出问题，做到只划“句号”、不划“逗号”，做到讲实话、干实事，言必信、行必果。加大经济环境治理力度，加快社会信用体系建设，巩固扩大“两集中、两到位”成果，全面规范并联审批，深入推进为民服务全程代理。严格政府绩效管理，加大治慵治懒治散力度，营造公平、高效、有序的发展环境。

（四）加强廉政建设，提升拒腐防变能力。严格落实党风廉政建设责任制，以坚决的态度、改革的举措，把政府系统廉政建设和反腐败工作不断推向深入，做到干部清正、政府清廉、政治清明。认真执行领导干部报告个人事项等制度，深入推进廉政风险防控管理，加强行政监察和审计监督，完善公共资源交易管理制度，把权力关进制度的笼子。严格执行中央八项规定、国务院“约法三章”和省市相关规定，进一步精简会议和文件，减少事务性应酬。厉行勤俭节约，严格控制“三公”经费支出和表彰庆典活动。坚持以零容忍态度惩治腐败，严肃查处各类违法违纪案件，切实做到有腐必反、有贪必肃。各级政府工作人员尤其是领导干部要始终牢记“两个务必”，时刻保持蓬勃朝气、昂扬锐气、浩然正气，带头做廉洁自律、勤政为民的表率！

各位代表!百舸争流千帆竞发，奋勇当先时不我待！让我们紧密团结在以习近平同志为核心的党中央周围，在市委的坚强领导下，紧紧依靠全市人民，励精图治，攻坚克难，锐意进取，为全面开创阜阳经济社会发展的新局面而努力奋斗！
